# Use of Thermal Black as Eco-Filler in Thermoplastic Composites and Hybrids for Injection Molding and 3D Printing Applications

**DOI:** 10.3390/molecules25071517

**Published:** 2020-03-26

**Authors:** Mihaela Mihai, Karen Stoeffler, Edward Norton

**Affiliations:** 1 Polymer Bioproducts, Advanced Manufacturing, Automotive and Surface Transportation Research Center, National Research Council Canada, 75 De Mortagne, Boucherville, QC J4B 6Y4, Canada; Karen.Stoeffler@cnrc-nrc.gc.ca; 2 Cancarb Limited, 1702 Brier Park Crescent N.W., Medicine Hat, AB T1C 1T9, Canada; edward_norton@cancarb.com

**Keywords:** thermal black, polypropylene composites, polyamide composites, acrylonitrile-butadiene-styrene composites, polyphenylene sulfone composites, thermoplastic hybrids, injection molding, 3D printing

## Abstract

Thermal black (TB) is one of the purest and cleanest forms of carbon black (CB) commercially available. TB is manufactured by the decomposition of natural gas in the absence of oxygen while the common furnace CB is derived from the burning of organic oil. TB has a larger particle size, a lower surface area, and lower level of particle aggregation, while being the most eco-friendly grade among the CB family. This study is the first-time evaluation of TB as filler in composites and hybrids based on thermoplastics such as polypropylene (PP), polyamide 6 (PA6), polyphenylene sulfide (PPS), and acrylonitrile butadiene styrene (ABS). TB loadings in composites were varied from 1 up to 40 wt. % and, in hybrids, the TB was used in combination with carbon fibers (CFs) at total contents up to 20 wt. %. TB-containing composites and hybrids based on PA6 and ABS were also extruded in filaments, used in 3D printing, and the obtained 3D printed parts were characterized. TB provided a very high loadability in thermoplastics while preserving their viscosity and performance. TB can replace a fraction of expensive CFs in composites without important changes in the composites’ performance. The composites and hybrids exhibited electrical resistivity and good mechanical and thermal properties when compared to commercial compounds, while enabling significant cost savings. TB also showed to be an excellent coloring agent. TB proved to be an outstanding eco-filler for compounds to be used in injection molding and 3D printing technologies.

## 1. Introduction

The global CB market, evaluated at around 15 billion USD, is currently one of the most mature. In terms of CB consumption, the tire industry accounts for 70% and the rubber industry accounts for 20% with the remaining of 10% being used in numerous niche applications [1,2]. The terminology *carbon black* refers in a generic way to many grades of commercial CB manufactured by different methods. The most common manufacturing method is the furnace black process. It consists in burning aromatic oils in a reactor to form CB particles and a tail gas mixture from which the CB is separated, densified, and processed into pellets of a variety of grades and sizes. The regular furnace black manufacturing process requires between 1.8 and 2.5 tons of oil to produce one ton of CB [3,4]. The resultant tail gas contains CO_2_, CO, sulphur compounds, CH_4_, and non-methane volatile organic compounds. A portion of this tail gas is generally burned for energy recovery, while the other portion is vented uncontrolled to the atmosphere [5]. Furnace black manufacturers emit around 38 million tons of CO_2_ per year. Approximately 95% of worldwide CB production is by way of the furnace black process and the remaining 5% is produced by way of other processes [3,4]. The oldest manufacturing process of CB, the lampblack process, consists in heating aromatic oils in an air-open flat cast-iron pan and provides CBs with broad particle size distribution from 60 to over 200 nm while the entire tail gas is released in the atmosphere. In the acetylene black process, acetylene is the raw material which is burned to form unpelletized CB powders [6,7]. Compared to the aforementioned processes, the TB manufacturing process is more eco-friendly. The TB is produced by the decomposition of natural gas at around 1400 °C in the absence of air or flame. This process produces TB particles and H_2_ using a two-reactor unit in which the reactors alternate between TB production and reheating. The reheating of a reactor is achieved by recirculating the hot H_2_ from the second reactor. The remainder of the high temperature H_2_ is redirected to produce high-pressure superheated steam which further is used to generate electricity [8]. The use of waste heat to generate electricity offsets other carbon-based power sources (coal and natural gas) used in the geographic vicinity. TB and CB belong at the same family of nano-fillers and are expected to fingerprint the properties of a material in the same manner, despite the differences in their manufacturing processes. 

Currently, furnace CB, due to its higher tinting strength compared to iron black or organic pigments, is widely used as a coloring agent for ink and paints but also for thermoset and thermoplastic materials and parts. CB provides resistance against UV radiation when just a small amount is mixed with resins which justify its utilization in parts for automotive applications such as bumpers, wire coverings, and pipe linings which require weather resistance. Moreover, materials produced from the mixture of CB with thermoplastics, elastomers, paints, adhesives, and pastes present excellent electrical conductivity because of the graphite-type paracrystalline structure of CB. For example, automotive parts such as fuel caps or fuel-introducing pipes are asked to be electrically conductive to prevent static buildup, therefore the CB also plays the role of antistatic agent [3,4]. 

An important body of scientific literature exists on the use of CB manufactured by the furnace process as filler in different thermoplastic polymers. Several studies concentrate on its main utilization as conductive filler in PP based compounds. It was stated that a PP compound becomes conductive at a percolation threshold of CB of 1.4 vol%, which is advantageously lower when compared to similar carbon-based fillers such as carbon nanotubes (threshold observed at 2.1 vol%) and synthetic graphite particles (threshold observed at 13 vol%) [9]. The conductivity of PP/CB compounds was observed to be reached more rapidly when a second carbon-based filler is used in combination with CB particles. This phenomenon was explained by a synergistic-type interaction between the two carbon fillers [10,11,12]. In another study, PP/CB conductive composites with a very low percolation threshold of 0.37 vol% were obtained by constructing a particular continuous segregated structure [13]. CB is also an excellent pigment for PP at very low concentrations. It is considerably more effective in absorbing light than other materials. In general, extremely fine CB particles, of 10 up to 30 nm in diameter, found uses in black automotive paints and parts [14,15,16]. In terms of mechanical properties of PP/CB compounds, the CB presence does not reduce the mechanical performance of the PP. The increase of CB content in PP from 1 to 30 wt. % resulted in an increase of 10% in tensile strength (from 33 up to 37 MPa), and an increase of 40% in tensile modulus (from 600 up to 1100 MPa). In the same time, increasing CB content from 2 to 5 wt. % abruptly decreased the deformation at break from 900% down to 10% since the addition of CB restricted the motion of the polymer chains [17]. The same study showed that increasing the amount of CB increased also the thermal stability, and the temperature of thermal decomposition was shifted to higher temperatures, i.e., from 400 °C for pristine PP up to about 530 °C for PP composite containing 30 wt. % of CB. Fewer studies are dedicated to the utilization of CB in composites based on PA6, ABS, PPS or other engineering thermoplastics and, therefore, scarce data exists concerning their mechanical, thermal, or electrical performance. For PA6/CB composites, in which the filler was chemically modified before processing, the percolation threshold for electrical conductivity was observed at 8 wt. % of CB [18]. This percolation threshold in PA6 was rather studied considering the PA6/PP blends, by taking advantage of the blend morphology [19,20]. The better interfacial adhesion between CB and PA6 forced the CB particles to selectively locate in PA6 domains and, at increasing CB content, a sea-island structure was converted to a co-continuous morphology. The highest electrical conductivity was observed at 8 wt. % of CB in PP/PA6 blend at a phase ratio of 70/30 when the two phases formed a co-continuous structure. When a multistage stretching extruder was used to produce PA6/PP/CB composites, the conductivity threshold of CB was decreased down to 1.5 wt. % [21,22]. Excepting the electrical conductivity, other properties of PA6 composites containing CB have been hardly studied. This is also the case for ABS/CB and PPS/CB composites. 

3D printing of thermoplastics by fused filament fabrication (FFF), is an effervescent and disruptive technology with a high potential to transform the industry and the product development process. This growing technology appeals to important innovation for new materials, technology progress, molds, and tooling making, but also direct manufacturing of small series parts. The thermoplastic filaments available on the market for FFF are in general based on PP, ABS, PA6, PLA, and some filaments based on high performance thermoplastics such as polyether imide (PEI) and polyether ether ketone (PEEK). Recently, the scientific community started to study the effect of different fillers and reinforcements in the thermoplastic filaments for application in 3D printing by layer-by-layer deposition of composite parts. The electrical resistivity of 3D printed parts using ABS/15 wt. % CB composite filaments was recently reported. It was observed that the electrical resistivity of the 3D printed parts was remarkably increased compared to the initial filaments. This electrical resistivity increment can be controlled by changing the 3D printing parameters used to construct the printed parts, such as the layer thickness, raster width, and air gap, a fact also observed by other published studies [23,24,25]. Unfortunately, as for thermoplastic/CB composites designed for extrusion and injection molding applications, there are no scientific papers disclosing the mechanical or thermal performance of thermoplastic/CB composite parts fabricated using 3D printing [26]. 

**Figure 1:**
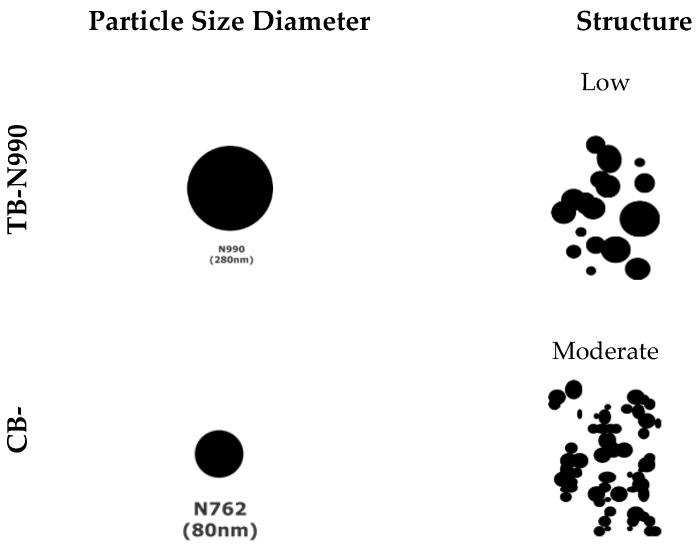
Dimensional and structural differences between Thermax^®^ N990 thermal black (TB) and Tokai^®^ N762 furnace carbon black (CB).

While considerable literature exists on composites containing CB, mostly studied for its innate electrical conductivity, to our best knowledge no scientific publications exist on composites containing TB. TB, like CB, has a great potential to be used as filler in many types of materials, including thermoplastics. The TB possesses the largest particle size among all carbon blacks and very low levels of particle aggregation while being one of the purest forms of carbon commercially available (Figure 1). Currently, TB finds some niches of applications as filler in rubbers (good dispersion, excellent processing etc.), in refractories (improved performance of carbon brick), in high temperature insulation (helps to handle the heat loss), in wires and cables (insulating properties due to its amorphous structure), in concrete (color retention, resistance to long-term weathering and aging), and in graphite production [27]. 

Even though the TB is considered to be an environmentally friendly grade from the CB family thanks to its manufacturing process, the TB did not find yet a well-deserved niche of application as a filler in thermoplastic polymers. As per our knowledge, there are no scientific publications on thermoplastics containing TB particles as filler designed for injection molding nor for 3D printing applications. This study is the first time evaluation of TB utilization in composites and hybrids based on different thermoplastics such as PP, PA6, PPS, and ABS. In the present work, the TB loadings in composites were varied from 1 up to 40 wt. % and in hybrids the TB was used in combinations with CF at overall contents up to 20 wt. %. Composites filaments containing 10 and 20 wt. % TB but also hybrids containing 10 wt. % TB/10 wt. % CF were designed for 3D printing by FFF. The mechanical and thermal properties, morphology, rheology, and electrical properties of composites were evaluated and disclosed with emphasis on the important effect of TB on the overall performance of obtained materials. This versatile eco-filler proves to be beneficial from many points of view when used in thermoplastic compounds and hybrids for injection molding and 3D printing applications. 

## 2. Results and Discussion

### 2.1. Thermoplastic Composites Containing TB 

#### 2.1.1. Morphology of PP, PA6, and PPS Composites

The pristine TB and CB, as resulting from their respective manufacturing processes, usually consist of chain-like clusters composed of spherical particles, so-called primary particles, but also of larger and tightly fused aggregates which form primary building blocks (Figure 1). The aggregates, being formed from elementary carbonaceous particles merged together at a temperature of about 1400 °C, cannot be broken up during the extrusion of composites, no matter the screw configuration used. 

**Figure 2:**
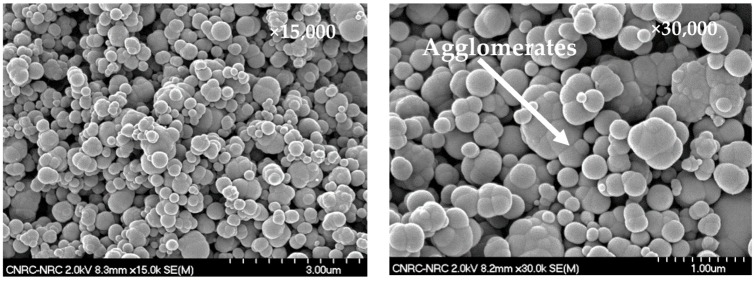
SEM morphology of pristine TB Thermax^®^ N990.

Figure 2 shows SEM micrographs at two different magnifications of the pristine TB N990, the TB grade used in this work. Its average diameter of particles was reported by the manufacturer to be 280 nm, which is much higher than the average diameter of a common CB, i.e., around 30 up to 90 nm. It was demonstrated that TB N990 presents on its surface only carbon (98.1%) and oxygen (1.9%) as elements. In addition, the furnace CB also presents sulphur (0.8%) on its surface [28,29]. The absence of sulphur in the case of TB N990 was explained by the purity of the feedstock used for its production. 

Moreover, the surface oxygen concentration of TB N990 was found to be higher compared to CB which should indicate that TB N990 possibly contains reactive oxygen sites. Therefore, when mixing it into a polymer melt at the extrusion temperature, TB may manifest affinity for chemical groups presenting polarity situated on the polymer backbone (such as for PA6, ABS, PLA, or other thermoplastic polymers containing functional groups).

In this work, the TB N990 was blended into thermoplastic polymer melts using an extrusion line having co-rotating screws which configuration was designed with the purpose to disperse and distribute the TB N990, i.e., its components such as the clusters and the aggregated blocks into PP, PA6, and PPS matrices (see process description and Figure 15 in the Materials and Methods section). 

**Figure 3:**
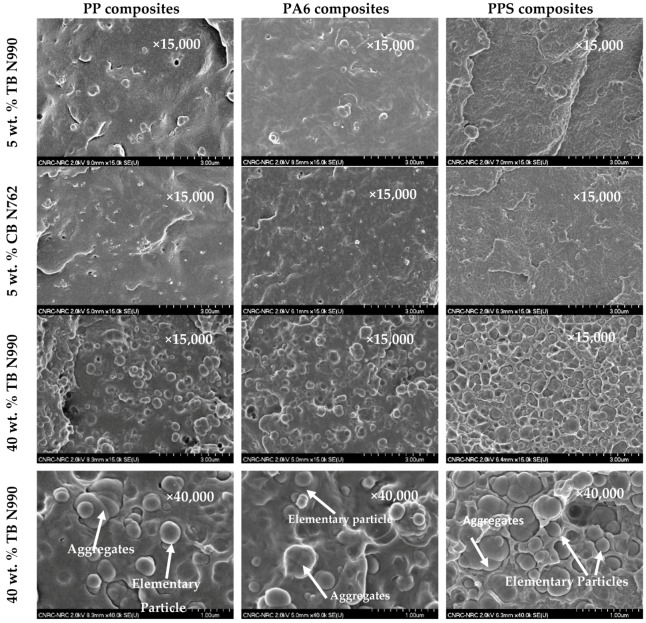
SEM micrographs of PP, PA6, and PPS compounds containing 5 wt. % TB N990 (1st row), 5 wt. % CB N762 (2nd row), and 40 wt. % TB N990 (3rd and 4th rows).

The formulations of the thermoplastic composites containing different concentrations of TB N990 and CB N762, the CB grade used in this work, are disclosed in Table 1. Figure 3 presents the microstructure of composites containing 5 and 40 wt. % TB N990 and 5 wt. % CB N762 observed on fractured surfaces as resulted from mechanical testing. Composites containing 1, 3, and 20 wt. % TB N990 were also observed in SEM but the micrographs were not shown here because of the similarity of observed trends. The TB N990 and of CB N762 demonstrated to be very well dispersed and distributed in each matrix. The difference in dimension of particles of TB N990 (about 280–300 nm) and of CB N762 (about 30–70 nm) is obvious from the presented micrographs. The microstructures of PP, PA6, and PPS composites containing 40 wt. % of TB N990 are presented on the 3rd row at a magnification of ×15,000 and on the 4th row at a magnification of ×40,000. The constituents of pristine TB N990, as observed previously in the Figure 2, i.e., the elementary particles and the fused aggregates, can be identified in the microstructures of PP/40 wt. % TB N990, PA6/40 wt. % TB N990, and PPS/40 wt. % TB N990 composites. 

A limited affinity is observed between the PP matrix and TB N990 particles. This affinity is probably due to both carbon-based structure of the PP and of the TB N990 (its surface contains 98.1% carbon) [28,29]. A good affinity is observed between TB N990 and the PA6 matrix because, as can be seen in the micrographs, the particles are covered by the PA6 matrix. This affinity may be supported, as discussed before, by the presence of oxygen (1.9%) on TB N990 surface. This oxygen, even in low concentration, possibly creates chemical bindings with the highly polar C=O and N-H groups on PA6 macromolecular chain. In the case of PPS/TB N990 composites, the presence of important voids between TB N990 particles and the matrix demonstrates a lack of affinity between those two phases. This may be explained by the impossibility to form chemical bridging between the oxygen from TB N990 surfaces and the aromatic rings (-C6H4-) linked by sulfides (-S-) of the PPS macromolecular chain. The aromatic rings -C6H4 - are recognized for their very low polarity which will be drawn by the -S- rather than by external O =, -O-, existing on TB N990 surface. It is very well known that a weak adhesion between the fillers and the matrix always leads to a drop of mechanical performance of the composites at increasing the filler concentration. Thus, PA6 and PP composites are expected to perform well in terms of mechanical properties, and the mechanical performance of PPS composites is expected to decline as the content of TB N990 increases.

#### 2.1.2. Rheology of PP, PA6, and PPS Compounds

**Figure 4:**
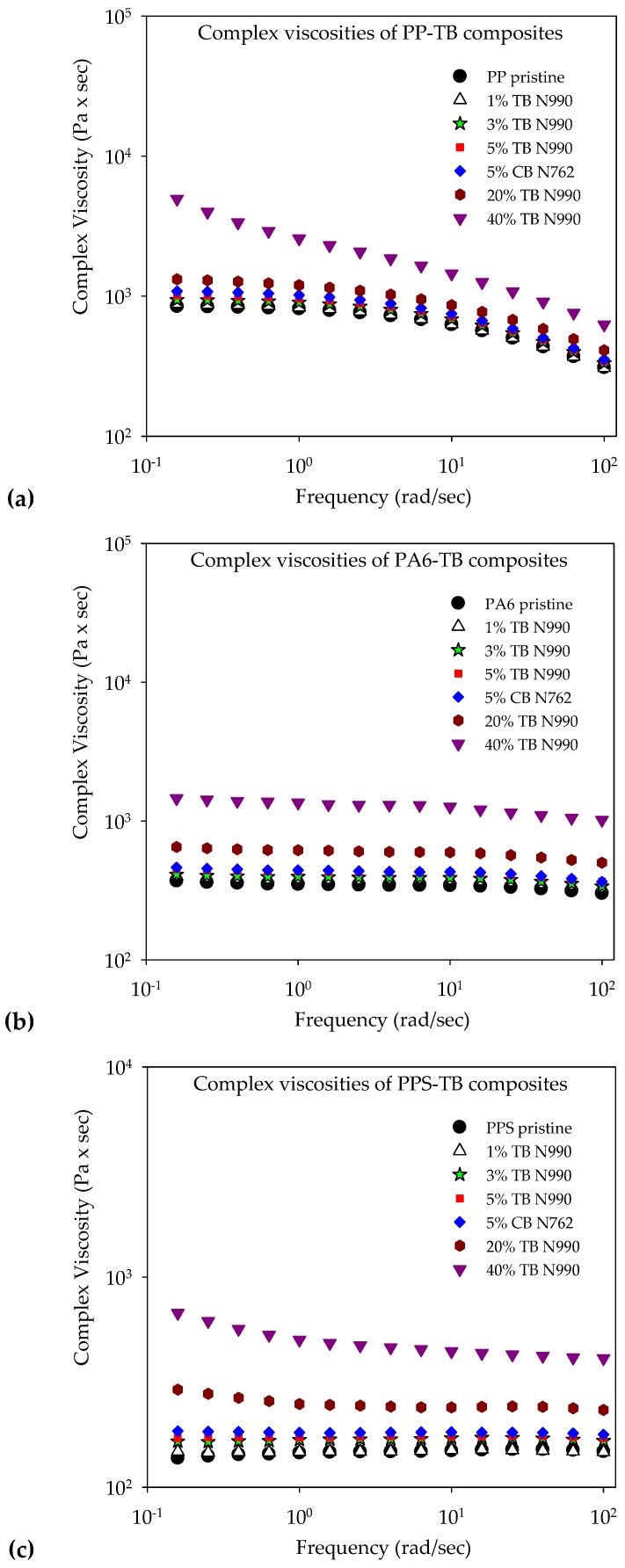
Complex viscosity curves of: (**a**) PP/TB N990, (**b**) PA6/TB N990, and (**c**) PPS/TB N990 composites.

The complex viscosity of PP/TB N990, PA6/TB N990 and PPS/TB N990 composites, of each pristine matrix, and of composites containing 5 wt. % CB N762 are disclosed in Figure 4a–c. The viscosities were evaluated at the same temperature as used in the extrusion process, i.e., at 200 °C for PP/TB N990 composites, 240 °C for PA6/TB N990 composites, and 300 °C for PPS/TB N990 composites. As can be seen from Figure 4a, the viscosity of PP/TB N990 compounds increased with TB N990 content. At low and medium concentrations of TB N990 of up to 20 wt. %, the viscosities remained near to that of pristine PP preserving similar Newtonian plateau at low frequencies and similar shear thinning behavior at higher frequencies. The PP/40 wt. % TB N990 shows a higher viscosity at low frequencies and a lack of Newtonian plateau. This suggests a possible formation of a rheological percolation threshold at this high TB N990 concentration, a fact also observed in the literature where the percolation threshold of CB in PP was stated to start at 19–21 wt. %. This rheological percolation threshold was supposed to be related to the electrical percolation threshold [30]. For the composites tested in this work, this should reflect further in an abrupt decrease in electrical resistivity at TB N990 concentrations somewhere from 20 up to 40 wt. %. The same rheological observations can be done concerning the behaviors of PA6/TB N990 composites (Figure 4b) and PPS/TB N990 composites (Figure 4c). At 1, 3, 5, and 20 wt. % TB N990, their viscosities remained very similar to those of pristine PA6 and PPS matrices. At 40 wt. % TB N990 in PA6 and PPS, a more accentuated increase in viscosity was observed, very similar to the one observed in PP, suggesting again the existence of percolation thresholds between 20 and 40 wt. % TB N990 in those matrices.

Overall, the PP, PA6 and PPS presented similar levels of increments in their complex viscosities, i.e., less than one order of magnitude in each case, at high filler concentration. It was demonstrated that the presence of spherical nanoparticles may decrease the viscosity by acting as plasticizers, especially at low-to-moderate interactions with the polymer [31,32]. For the studied composites, the effect of high concentrations of low-structured spherical TB nanoparticles on the viscosity seems to prevail over the low-to-moderate chemical interaction between each matrix and the oxygen existing on TB surface. CB N762, due to its higher level of structure, presented a faintly higher complex viscosity than the composite containing 5 wt. % TB N990. At loadings higher than 5 wt. %, this difference in viscosity should be more significant.

#### 2.1.3. Mechanical and Thermal Properties of PP, PA6, and PPS Compounds

**Figure 5:**
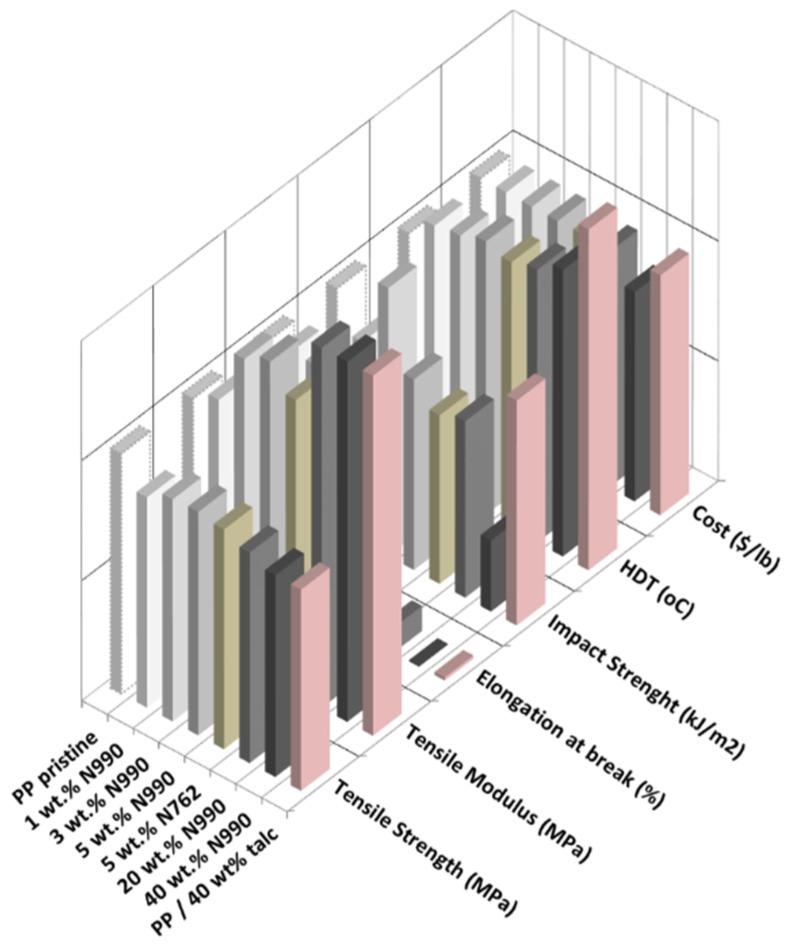
Comparison of tensile properties, impact properties, HDT, and materials costs of PP/TB N990 composites from this work and the PP/40 wt. % talc, a commercial automotive grade.

**Figure 6:**
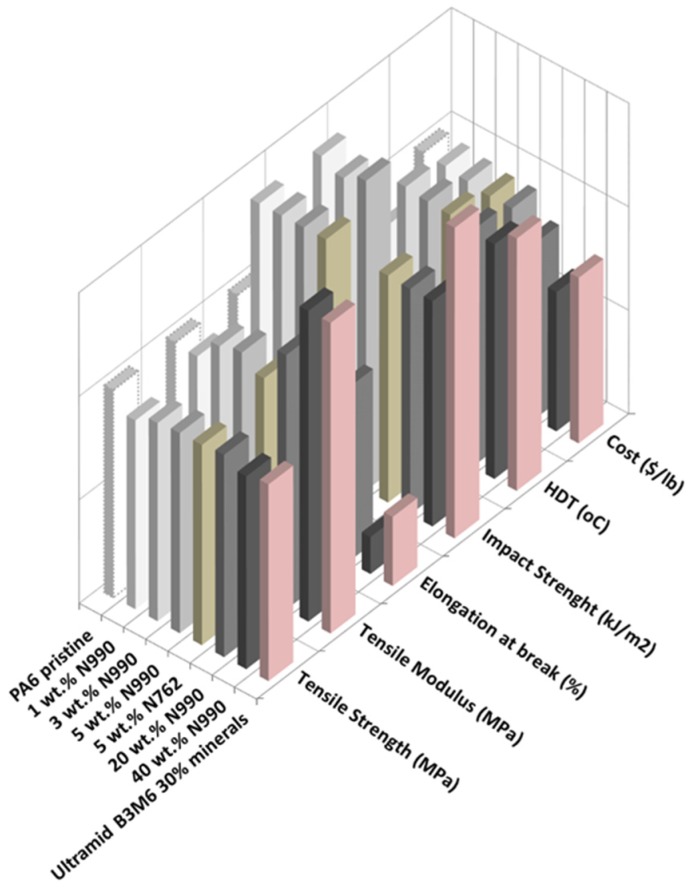
Comparison of tensile properties, impact properties, HDT, and materials costs of PA6/TB N990 composites from this work and the PA6/30 wt. % minerals, a commercial automotive grade.

**Figure 7:**
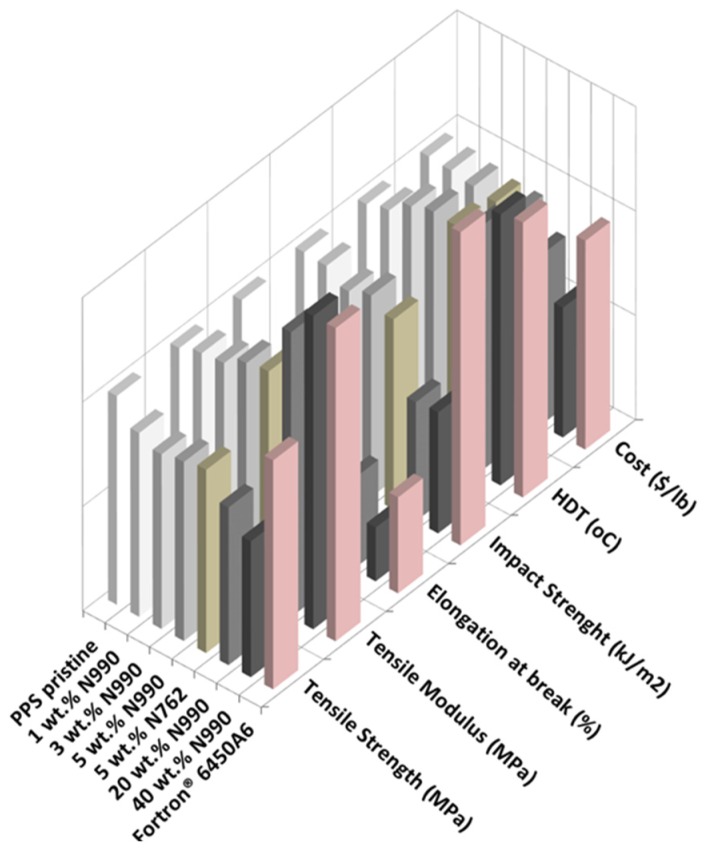
Comparison of tensile properties, impact properties, HDT, and materials costs of PPS/TB N990 composites from this work and PPS/40 wt. % minerals, a commercial automotive grade.

The mechanical and thermal performance as well as the estimated costs of PP/TB N990, PA6/TB N990, and PPS/TB N990 composites are disclosed in the 3D-column diagrams presented in the Figure 5, Figure 6 and Figure 7 (qualitative presentations) and their respective numerical values are disclosed in Table 2, Table 3, and Table 4 (quantitative presentations). Figure 5 and Table 2 unveil a comparison of tensile strength, tensile modulus, Izod impact strength, HDT, and calculated material cost of pristine PP matrix, PP composites containing 1, 3, 5, 20, and 40 wt. % TB N990, PP composite containing 5 wt. % CB N762, and a commercial grade PP/40 wt. % talc. The main observation is that increasing the TB N990 content in PP did not decrease the tensile strength (TS) of composites which remained similar to the one of pristine PP, i.e., around 30 MPa. This is an advantage of TB N990 use in PP composites compared to the usual behavior of a polyolefin containing mineral particles which typically leads to a decrease in TS, particularly at high loadings. A number of researchers have documented this phenomenon in various polyolefins, i.e., a TS or flexural strength (FS) reduction with increasing mineral filler content [33,34]. It was shown that the strength of HDPE/talc composites decreased with increasing talc concentration, but, when CB was used as filler in HDPE, the strength slightly increased with CB content. This was explained by a higher compatibility between a polyolefin and an organic filler (high carbon content), such as CB, compared to the one between a polyolefin and an inorganic filler, such as talc. A low compatibility negatively affects the performance of composites. In order to improve this compatibility, their interphase should be modified with a coupling agent. In this work, the high loadings of TB N990 in PP, such as 20 and 40 wt. %, did not bring changes in the TS of the composites compared to the PP matrix alone, even in the absence of a coupling agent. Thus, this supports the SEM observations, as disclosed in the Figure 3, on the existence of a potential low-to-moderate affinity between the PP matrix and the TB N990 organic particles. 

The tensile modulus (TM) of PP/TB N990 composites highly increased with TB concentration. The TM increment was about 28% for PP/5 wt. % TB N990, 67% for PP/20 wt. % TB N990, and about 147% for PP/40 wt. % TB N990 (Table 2). The value of the elongation at break at low TB N990 concentrations was preserved compared to pristine PP (around 900%) and abruptly decreased at 20 wt. % TB N990 content (down to 95%).

As expected, the Izod impact strength (IS) of the pristine PP of 3.2 kJ/m^2^ was decreased down to around 2.5 kJ/m^2^ at low and medium TB N990 contents and for PP/40 wt. % TB N990 was decreased down to 1 kJ/m^2^. The HDT of pristine PP, 88 °C, increased with TB N990 content up to 105 °C for PP/40 wt. % TB N990. The recommended HDT for PP composites designated for interior automotive applications is 90 °C or higher. The PP/TB N990 composites obtained in this work may be recommended for this type of application. It can be also concluded that the TB N990 performed similarly to the CB N762 in PP and the performance of PP/40 wt. % N990 composite compares well to the commercial grade PP/40 wt. % talc. Another advantage of the use of TB N990 in PP composites is a cost reduction which may be up to 13%. 

Comparisons of TS, TM, Izod IS, HDTs, and materials costs of pristine PA6 matrix, PA6 composites containing 1, 3, 5, 20, and 40 wt. % TB N990, PA6/5 wt. % CB N762, and commercial grade PA6/30 wt. % minerals are presented in the Figure 6, and the numerical values can be found in Table 3. The TS of PA6/TB N990 composites presented slightly lower values (i.e., 73–78 MPa) than pristine PA6 (i.e., 80 MPa), but, overall, those TS values remained in the same range as for pure PA6 even at the high loading of 40 wt. % TB N990. The lack of deterioration of TS of the PA6 composites with increasing TB N990 content up to 40 wt. %, even in the absence of a coupling agent, seems to point to a good adhesion between PA6 and TB N990. This result confirms the morphological observations presented previously in Figure 3. Therefore, high loadings of TB N990 can be used in PA6 without significant changes in TS. As expected, the TM increased with TB N990 content. At low concentrations of TB N990, from 1 up to 5 wt. %, the TM slightly increased compared to pristine PA6, but at 20 wt. % TB N990 the TM increased by 23% and at 40 wt. % TB N990 the TM increased by 62%. The elongation at break remained in the same range as for pristine PA6, i.e., for low and for high TB N990 concentrations in PA6. The Izod IS of pristine PA6 (2.4 kJ/m^2^) was increased with TB N990 addition, which is also an indication of a potential low-to-moderate good adhesion between the matrix and the fillers. At low TB N990 concentrations of 1 up to 5 wt. % the IS increased by around 50% reaching values of 3.4–3.6 kJ/m^2^ and at high TB N990 contents decreased down to 2.6 kJ/m^2^. The HDT of pristine PA6, 160 °C, increased with the addition of TB N990 up to 181–189 °C, i.e., an increment of about 14%. As for PP compounds, PA6/5 wt. % TB N990 and PA6/5 wt. % CB N762 presented very similar thermal and mechanical properties. The mechanical and thermal performance of the PA6/40 wt. % TB N990 compares very well to commercial PA6/30% minerals having automotive approvals. The material cost reduction of PA6/TB N990 composites increased with the TB N990 content, from 0.75% when 1 wt. % of TB N990 was used up to 34% when 40 wt. % TB N990 was used in PA6 (Table 3). Moreover, these important reductions of materials costs come with a preservation or a slight increment in mechanical and thermal properties of composites. For that reason, PA6/TB N990 composites could replace, for example, the PA6 commercial compounds used in interior automotive applications. 

Property comparisons of PPS, PPS composites containing 1, 3, 5, 20, and 40 wt. % TB N990, PPS/5 wt. % CB N762, and a commercial grade PPS/fiberglass/minerals, in terms of TS, TM, Izod IS, HDT, and material cost, are presented in Figure 7 and the numerical values can be found in Table 4. In contrast to the mechanical performance of PP/TB N990 and PA6/TB N990 compounds discussed previously, the TS, the elongation of break, and the Izod IS of PPS/TB N990 compounds decreased with TB content. The TS decreased continuously from 82.1 MPa for pristine PPS down to 53.4 MPa for PPS/40 wt. % TB N990 while the elongation at break decreased from 3.3 down to 0.8%. The Izod IS of pure PPS decreased from 2.7 kJ/m^2^ down to 1.5 kJ/m^2^ for high TB N990 contents in PPS.

This decrease in mechanical performance can be explained by a lack of affinity between PPS matrix and TB N990 particles as formerly concluded by the morphological observations (Figure 3). On another hand, PPS is a brittle polymer and the addition of TB N990 particles increased the brittleness of PPS composites. As expected, the TM of PPS/TB N990 compounds increased with the filler addition reaching a 36% increase for PPS/20 wt. % TB N990 and a 93% increase for the PPS/40 wt. % TB N990 composites. An important improvement was observed for the thermal performance of PPS/TB N990 composites. The HDT increased with TB content, from 167 °C for pristine PPS up to 217 °C for PPS/40 wt. % TB N990 which is a higher temperature than recommended for under the hood utilization (i.e., 205 °C). The PPS/5 wt. % TB N990 and PPS/5 wt. % CB N762 presented similar performances, but the PPS/TB N990 composites clearly need formulation improvements to reach the performance of the commercial PPS composite grades currently used in automotive parts applications, but PPS being an expensive polymer, the PPS/TB N990 compounds benefit from up to 40% cost reduction at high concentrations of TB. In the case of the utilization of an expensive matrix, such as PPS, the use of an inexpensive filler, such as TB N990, may greatly benefit from a reduction of the cost while the compound can still be used for its higher thermal performance and its high tensile modulus compared to the pristine PPS.

#### 2.1.4. Electrical Conductivity of PP, PA6, and PPS Compounds

CB particles, as presented in the Introduction, are used in different types of materials to increase the electrical conductivity. This innate electrical conductivity of CB and of its composites is induced by the graphite-type paracrystalline structure of the CB particles, by its high level of structure, and by the formation of paths of CB particles at low-medium contents in the polymer.

**Figure 8:**
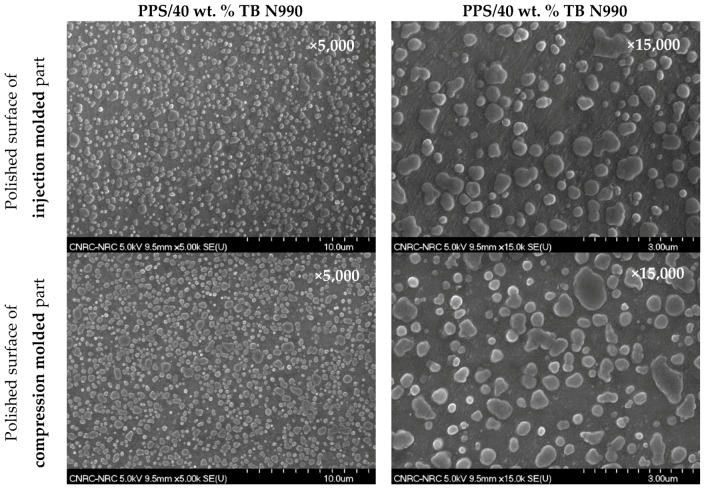
SEM morphologies of polished surfaces of injection molded (1st row) and compression molded (2nd row) of PPS/40 wt. % TB N990 compounds.

In this work, the PP/40 wt. % TB N990, PA6/40 wt. % TB N990, and PPS/40 wt. % TB N990 composites were selected for electrical conductivity testing because of their highest TB N990 contents. Electrical testing for those three composites was done using different types of specimens and current intensities, i.e.: (1) injection molded discs for a current intensity (I) of 5 A, (2) injection molded discs polished on their surface, and I = 5 A, (3) polished injection molded discs and I = 10 A and (4) compression-molded discs coalesced at adequate temperatures for 10 minutes and polished on the surface for I = 5 A. The sample surfaces were polished for some tests with the purpose to expose the specimen interior by removing the thin layer of polymer which forms at the surface during the injection molding process. Figure 8 presents the morphology of a polished surface of an injection molded specimen (1st row) and a polished surface of a compression molded specimen (2nd row) for PPS/40 wt. % TB N990. The PP/40 wt. % TB N990 and PA6/40 wt. % TB N990 specimens presented identical morphologies and, for simplicity, those SEM images were not presented here. 

All tested specimens were found to be highly electrically resistive for all applied tests. By opposition to CB, no pathways of TB N990 particles seems to be formed in PPS/40 wt. % TB N990 injected or compressed specimens, as can be observed from the Figure 8. This lack of electrical conductivity can be supported also by larger particle size, the lower level of structure of TB N990 (Figure 1 and Figure 2) and by the excellent dispersion of TB N990 particles during the extrusion (Figure 3). Moreover, the surface oxygen concentration of TB N990 was found to be higher compared to CB [28,29]. It was demonstrated that the electrical conductivity decreases with increasing surface concentration of non-carbon. This might further explain the absence of electrical conductivity of TB particles themselves. In conclusion, the electrical resistivity of PP/40 wt. % TB N990, PA6/40 wt. % TB N990, and PPS/40 wt. % TB N990 composites, indicates that the ones containing less than 40 wt. % TB N990 are also electrically resistive. This can be an important advantage of the use of TB N990 as fillers in non-electrically conductive composites designated for automotive interior parts, such as pillars, dashboards, door panels, central consoles, and others.

**Figure 9:**
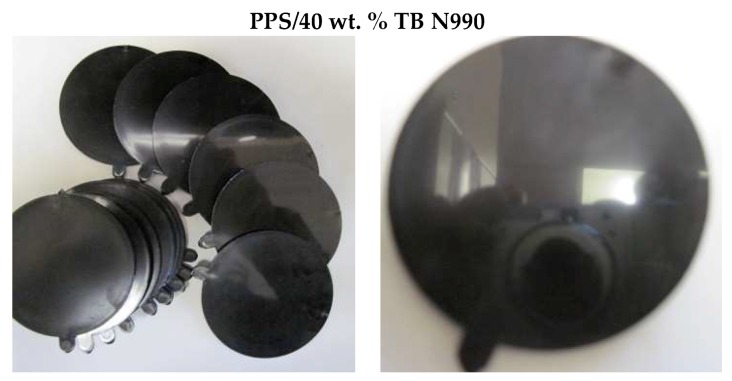
Physical aspect of injection molded specimens based on PPS/40 wt. % TB N990 composite.

Figure 9 presents the physical aspect of injected parts based on PPS/40 wt. % TB N990 composite. Similar physical aspects were observed also for lower TB contents in the polymer matrices. The injected samples have high-quality mirror-like surfaces, very similar to so-named “piano black” surfaces used by automotive industry. Normally, high-gloss appearance of injected automotive parts may be obtained by applying paints or by using coatings based on polycarbonate (PC) of unpainted polymethyl methacrylate (PMMA). In our case, the TB gave rise to the brilliant shade surfaces without using a supplementary treatment step. The injection molding process and parameters to be applied to TB containing thermoplastic composites has to be further improved, but, at this stage, the “piano black” look given by the presence of TB particles is competing with PMMA and PC used in automotive applications but at much lower cost.

### 2.2. Thermoplastic Hybrids Containing TB/CF

Limited scientific literature exists describing the effect of the combination of two types of carbon-based fillers in hybrid thermoplastic composites manufactured through melt processing and injection molding. Some synergies were observed and were investigated from the point of view of electrical conductivity performance of the hybrids. One review on this topic containing more details can be found in the reference [35]. This work is the first-time evaluation of performances of hybrid composites containing a combination of TB N990 and CF, a carbon-based filler and a carbon-based fiber. The formulation of the thermoplastic hybrids containing combinations of CF/TB N990 were disclosed further in Table 5.

#### 2.2.1. Morphology of TB/CF Hybrids Based on PP, PA6, and PPS

**Figure 10:**
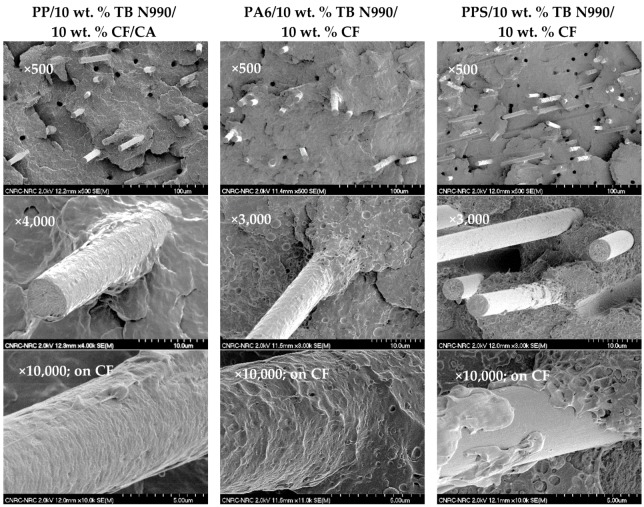
SEM micrographs of fractured surfaces for: the hybrid PP/10 wt. % TB N990/10 wt. % CF/CA-1st column, the hybrid PA6/10 wt. % TB N990/10 wt. % CF-2nd column, and for hybrid PPS/10 wt. % TB N990/10 wt. % CF-3rd column.

Figure 10 shows SEM micrographs obtained on fractured surfaces resulting from mechanical testing of hybrid specimens based on PP, PA6, and PPS matrices containing 10 wt. % TB N990/10 wt. % CF. The micrographs corresponding to the hybrid PP/10 wt. % TB N990/10 wt. % CF/CA are presented in the 1st column, to the hybrid PA6/10 wt. % TB N990/10 wt. % CF in the 2nd column, and to the hybrid PPS/10 wt. % TB N990/10 wt. % CF in the 3rd column. The micrographs of hybrids containing 17 wt. % CF/3 wt. % TB N990 and 15 wt. % CF/5 wt. % TB N990 showed similar microstructures and are not disclosed here. The micrographs are presented from the top to the bottom at increasing magnifications.

The micrographs disclosed in the 1st row show a good dispersion of the CF in all three matrices. In the micrographs presented in the 2nd row, at higher magnification, it can be observed that an affinity exists between the CF and PP matrix and CF and PA6 matrix which explains the polymer covering the CF surfaces. Obviously, it was not the case for CF and PPS matrix which seem to lack affinity as disclosed in all micrographs presented in the 3rd column (as also observed in the Figure 3). The 3rd row presents micrographs of one CF surface in the hybrids at a higher magnification. In the case of PP/10 wt. % TB N990/10 wt. % CF/CA hybrid, the presence of CA was very beneficial for the adhesion between CF and PP matrix and led to a complete covering of CF and the TB particles by PP matrix. The TB N990 particles seem also to be very well anchored on the examined CF surface. A good adhesion has helped in the same manner in the case of the hybrid PA6/10 wt. % TB N990/10 wt. % CF where the TB N990 particles were also well anchored on the CF surface. 

**Figure 11:**
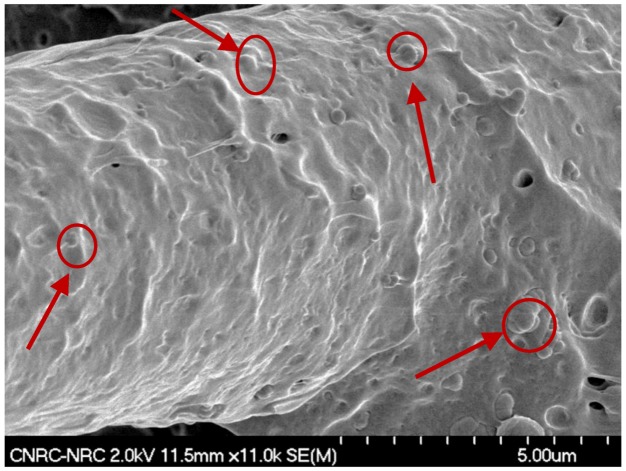
SEM micrograph obtained on one CF surface covered by the polymer matrix and TB N990 particles (in PP and PA6 hybrids only).

This observation for PP and PA6 hybrids is demonstrated in the Figure 11. This phenomenon of TB N990 particles gripped on CF surfaces might strengthen the CF reinforcement effect and might be further reflected in the mechanical performance of the hybrids.

#### 2.2.2. Rheology of TB/CF Hybrids Based on PP, PA6, and PPS

**Figure 12:**
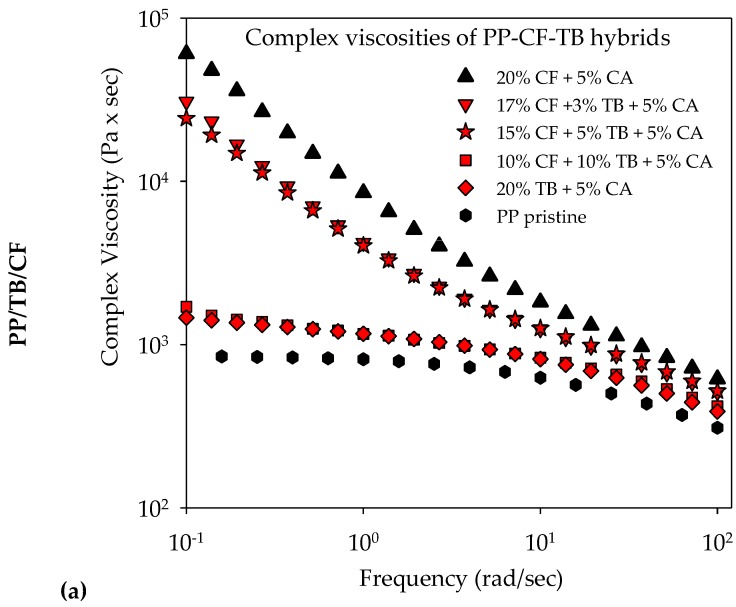

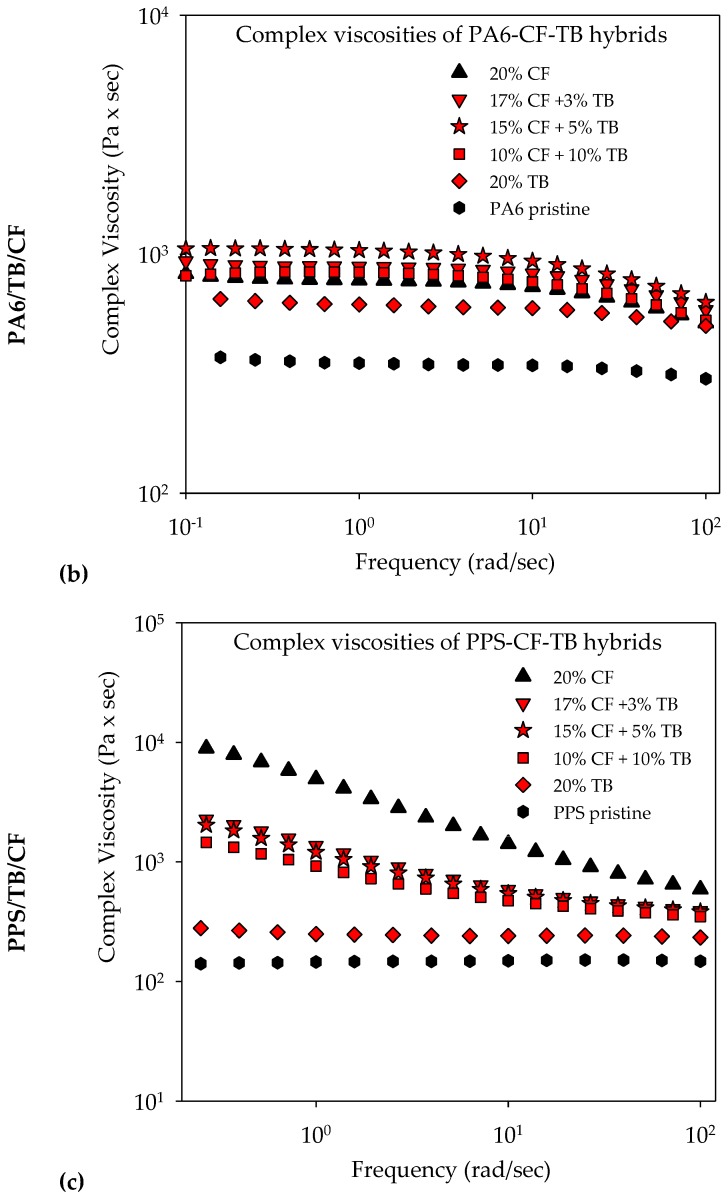
Viscosity curves of: (**a**) PP/TB N990/CF, (**b**) PA6/TB N990/CF, and (**c**) PPS/TB N990/CF hybrids.

Figure 12 unveils the rheological behaviors of CF/TB N990 hybrids based on PP, PA6, and PPS respectively. The complex viscosities of pristine PP, PP/20 wt. % CF, PP/20 wt. % TB N990, and PP hybrids are presented in the Figure 12a, of pristine PA6, PA6/20 wt. % CF, PA6/20 wt. % TB N990, and PA6 hybrids are presented in the Figure 12b, and of pristine PPS, PPS/20 wt. % CF, PPS/20 wt. % TB N990, and PPS hybrids are presented in the Figure 12c. As discussed previously (see Figure 4), the addition of 20 wt. % TB in each matrix did not have an important impact on the viscosity of pristine PP, PA6, or PPS. Contrary to TB N990, CF at the same content of 20 wt. % highly increased the viscosities of the matrices as can be observed in the Figure 12a–c respectively. It can be observed from the Figure 12a that in the case of PP hybrids, the complex viscosities of PP/17 wt. % CF/3 wt. % TB N990 and of PP/15 wt. % CF/5 wt. % TB N990 are only slightly lower than the one of PP/20 wt. % CF. 

From the Figure 12b, it can be observed that all PA6/CF/TB N990 hybrids have similar complex viscosities as PA6/20 wt. % CF. Finally, in the Figure 12c, it can be observed that PPS/CF/TB N990 hybrids have complex viscosities about one order of magnitude lower than PPS/20 wt. % CF and about one order of magnitude higher than PPS/20 wt. % TB N990. Therefore, an interesting phenomena can be observed for the PP/20 wt. % CF and PA6/20 wt. % CF composites: when up to 10 wt. % of CF are substituted by an equal content of TB N990, the rheological behavior of the hybrids remained comparable to the one of 20 wt. % CF containing composites. This phenomenon was not observed for PPS based hybrids. Usually, an interrelation exists between the complex viscosities of thermoplastic composites (i.e., their flow behavior at the compounding temperature), their microstructure, and their mechanical performance (i.e., their behavior at ambient utilization temperature). As presented previously in the Figure 10 and Figure 11, the morphological microstructures of PP/CF/TB N990 and PA6/CF/TB N990 hybrids presented particles of TB N990 attached on the surface of CF. Therefore, it should be assumed that TB N990 particles may strengthen the CF presence in the matrix. It seems that, at some level, a potential synergy may exist between the components of the PP and PA6 hybrids which is reflected in their rheological behavior and, further, might be reflected in their mechanical performance.

#### 2.2.3. Mechanical and Thermal Properties of TB/CF Hybrids Based on PP, PA6, and PPS

Tensile properties, Izod impact values, and heat deflection temperatures of PP, PA6, and PPS hybrid composites and those of pristine matrices are presented in Table 6. The results disclose important increments of the TS of PP/20 wt. % CF and of PP/CF/TB N990 hybrids at the addition of the CA. The CA presence increased the TS of PP/20 wt. % CF from 34.4 to 80 MPa (a 250% increase compared to PP), the TS of PP/17 wt. % CF/3 wt. % TB N990 hybrid increased from 37 up to 74 MPa (a 240% increase compared to PP), and the TS for PP/15 wt. % CF/5 wt. % TB N990 hybrid increased from 38 up to 71.3 MPa (a 230% increase compared to PP). Surprisingly, the TS of PP hybrids are only slightly lower than the TS of the PP/20 wt. % CF/CA composites. The hybrid PP/10 wt. % CF/10 wt. % TB N990/CA presented a TS of 59.7 MPa, i.e., only 25% lower than the one of PP/20 wt. % CF/CA composites and 190% higher than PP. The PP/20 wt. % TB N990/CA disclosed a TS of 31.7 MPa. In terms of TM, the PP hybrids obtained in this study presented the same variations as the TS. The TM of PP/20 wt. % CF/CA of 9000 MPa (812% vs. PP) was slightly decreased down to 8050 MPa (732% vs. PP) and down to 7250 MPa (658% vs. PP) when 3 wt. % and 5 wt. % of CF were replaced by an equal quantity of TB N990. The PP/20 wt. % TB N990/CA composites presented the lowest TM of 1860 MPa. The elongation at break of PP/20 wt. % CF/CA of 8.3% was slightly increased up to 8.5, 9.3 and 11.1% when the CF were substituted by the 3, 5 and 10 wt. % TB N990. In terms of HDT, all PP hybrids presented values of 150–156 °C which were higher compared to the pristine PP (88 °C), to PP/20 wt. % TB N990/CA (108 °C), and compared to PP/20 wt. % CF/CA (148 °C). 

These important mechanical and thermal properties of PP/CF/TB hybrids, very similar compared to PP/20 wt. % CF, can be supported by the increased affinity due to the CA presence but also by TB N990 particles bonded on CF surfaces as observed previously from hybrid microstructures (Figure 10 and Figure 11). In conclusion, it is very advantageous to replace up to 10 wt. % CF by TB N990 in PP/20 wt. % CF because of the conservation of mechanical and thermal performances while decreasing the cost due to the lower price of TB N990 compared to the one of CF (Table 7). 

Table 6 presents also that the TS of pristine PA6 (i.e., 80 MPa) increased by 181% for PA6/20 wt. % CF (i.e., 145 MPa). Further, the TS of PA6/17 wt. % CF/3 wt. % TB N990 hybrid presented a value of 147 MPa (increase of 183% compared to PA6), the TS of PA6/15 wt. % CF/5 wt. % TB N990 hybrid presented a value of 135 MPa (increase of 168% compared to PA6), and the hybrid PA6/10 wt. % CF/10 wt. % TB N990 presented a value of TS of 114 MPa (increase of 143% compared to PA6). The TM of the hybrids varied in the same manner as the TS. When replacing the CF by TB N990 in PA6/20 wt. % CF, the TM changed from 11,400 MPa (381% compared to PA6) to 11,500 MPa (386% compared to PA6), to 10,300 MPa (343% compared to PA6) and down to 8200 MPa (274% compared to PA6) corresponding to 3, 5, and 10 wt. % TB N990 contents in the hybrids. The elongation at break of all the hybrids presented values of 75% from the pristine PA6. The IS of PA6/20 wt. % CF decreased from 4.4 kJ/m^2^ down to 3.7 and 2.6 kJ/m^2^ as the TB N990 content in the hybrids increased. Therefore, replacing the CF in PA6/20 wt. % CF composite by up to 10 wt. % TB N990 did not change significantly its mechanical properties. As seen before, a low-to-medium degree of compatibility exists between PA6 matrix, TB N990, and the CF, even in the absence of a CA, which may explain the disclosed mechanical proprieties. As observed before from SEM morphology (Figure 10 and Figure 11), and similarly to PP hybrids, the anchorage of TB N990 particles through the PA6 matrix on the surface of CF potentially coupled the reinforcement effect of the CF. The HDT of PA6 of 160 °C increased to 216 °C for PA6/20 wt. % CF and slightly decreased down to 214 and 210 °C for the hybrids containing 3, 5, and 10 wt. % TB N990. Therefore, compared to PA6/20 wt. % CF composite, the PA6/CF/TB N990 hybrids present very similar mechanical and thermal properties as CF composites while having much lower costs (Table 7). The PA6/CF/TB hybrids present high utilization potential, for example, in automotive interior parts. 

The tensile properties, Izod impact strength, and heat deflection temperatures of PPS composites and hybrids are shown also in Table 6. The TS of PPS/20 wt. % CF composites, i.e., 151 MPa or 184% higher than the pristine PPS, slightly declined at the replacement of CF by 3, 5, and 10 wt. % TB N990 down to 145, 131 and 105 MPa respectively. The TM of PPS/20 wt. % CF composites, i.e., 20,000 MPa and 550% higher than the pristine PPS, slightly decreased at the replacement of CF by 3, 5, and 10 wt. % TB N990 down to 17,200, 17,100 and 13,500 MPa, respectively. The elongation at break of all PPS composites and hybrids remained around the pristine PPS value, i.e., around 4% and is explained by the innate brittle character of PPS. The Izod IS of the hybrids presented values of 3–4 kJ/m^2^ which is similar to the one of PPS/20 wt. % CF and slightly higher than for pristine PPS. The HDT of PPS (167 °C) increased up to 275 °C for the composite PPS/20 wt. % CF and slightly declined down to 272–268 °C for the hybrids. Overall, the properties of PPS/CF/TB N990 hybrids, as in the case of PP and PA6 hybrids, remained similar to the one of the PPS/20 wt. % CF composites. In conclusion, the replacement of CF by 3, 5 and 10 wt. % TB N990 in PPS/CF composites is very advantageous from point of view of performance preservation but also for cost-effectiveness of these PPS hybrids (Table 7).

### 2.3. Thermoplastic Composites and Hybrids Containing TB for 3D Printing

#### 2.3.1. Morphology of Composites and Hybrids Based on PA6 and ABS for 3D Printing

Among the composites and hybrids disclosed and discussed previously, the PA6/TB N990 composites and PA6/TB N990/CF hybrids were selected for 3D printing application. This selection was done following the good performance of PA6-based composites and hybrids and because the PA6 is one of the most commercialized thermoplastics for 3D printing. Besides, the ABS was also selected to obtain additional composites and hybrids for 3D printing application. Therefore, for the work continuation, PA6 and ABS composites containing 10 and 20 wt. % TB N990 and their hybrids containing 10 wt. % TB N990/10 wt. % CF were extruded using the same extrusion line and extrusion parameters as used previously (Figure 15). The compositions of all thermoplastic composites formulated for 3D printing are disclosed in Table 8.

**Figure 13:**
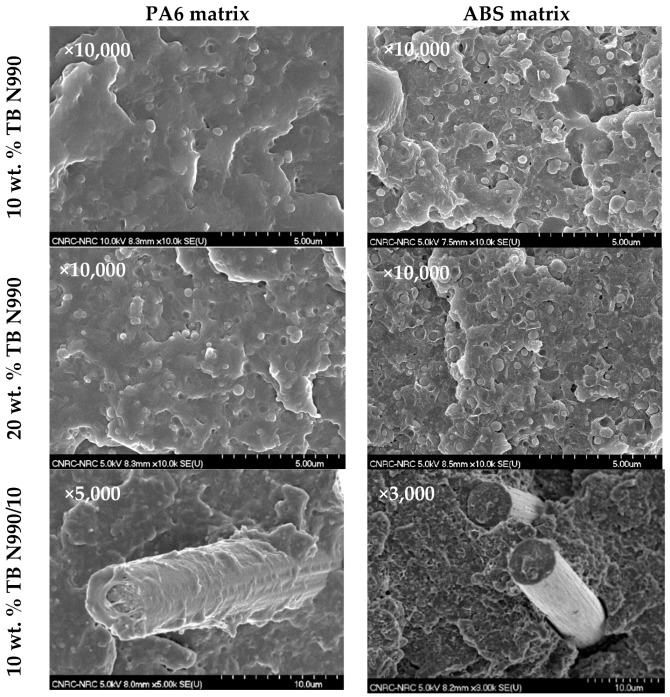
Micrographs of composites based on PA6 matrix (1st column), ABS matrix (2nd column), with 10 wt. % TB N990 (1st row), 20 wt. % TB N990 (2nd row), and 10 wt. % TB N990/10 wt. % CF (3rd row).

The obtained pellets of PA6 and ABS composites and hybrids were injected to obtain specimens for morphological observations and mechanical characterization. The morphologies of injected parts based on PA6 (1st column) and ABS (2nd column) containing 10 wt. % TB N990 (1st row), 20 wt. % TB N990 (2nd row), and 10 wt. % TB N990/10 wt. % CF (3rd row) are presented in the Figure 13. The shown micrographs were taken on fractured surfaces resulting from the Izod impact tests. All samples appear to have good dispersion and distribution of the TB N990 or TB N990/CF which is important in obtaining optimal properties. For PA6, as also stated previously, good interfacial adhesion can be observed between the matrix and the TB N990 and CF. In contrast, ABS shows only medium-weak interfacial adhesions between the matrix and fillers/fibers. 

#### 2.3.2. 3D printed Composites and Hybrids Based on PA6 and ABS

A certain level of flexibility of the filaments is needed for FFF application with the purpose to assure a continuity of printing without filaments breaking due to their rigidity and brittleness. The extruded filaments based on PA6 composites and hybrids presented high flexibility. The filaments extruded from ABS composites containing 10 and 20 wt. % TB N990 proved also to be sufficiently flexible for FFF, despite the innate brittle nature of the ABS. Only the ABS/TB/CF hybrid filament proved to be brittle during the 3D printing explained by its innate brittleness which was increased in the presence of carbon fibers. Therefore, the filament based on ABS/10 wt. % TB N990/10 wt. % CF hybrid was obtained again by extrusion with a low content of an elastomer added (Add) to its formulation. Therefore, all extruded PA6 and ABS filaments were used further in FFF to 3D print specimens for characterization. Specimens for tensile and Izod impact testing were printed for all the formulations. The parts were 3D printed completely filled so that their properties can be compared to injection molded parts of the same material. One observation which must be mentioned here is that the presence of TB N990 in composites and hybrids had an unexpected effect, i.e., it helped to increase the flexibility of the extruded filaments. This phenomenon might be explained by the spherical shape of TB N990 particles and their very low diameter. These spherical small TB N990 particles, once intercalated between the macromolecular chains during the extrusion, possibly assisted their sliding and, therefore, increased the flexibility of the composite filaments. 

#### 2.3.3. Properties of PA6 and ABS Injection Molded and 3D Printed Specimens

The mechanical performance of injection-molded and 3D printed specimens obtained from PA6 and ABS composites and hybrids containing 10 wt. % TB N990, 20 wt. % TB N990, and 10 wt. % TB N990/10 wt. % CF are compared in Table 9. The performance of the injected pristine polymers and 3D printed parts using commercial filaments are also included for comparison purposes. Table 9 unveils for the 3D printed parts based on PA6 and ABS composites and hybrids lower tensile properties than their injection molded counterparts. This is a normal reduction of properties and an explanation, among others, is the creation of empty spaces between the deposited layers during the 3D printing. These empty spaces, that cannot be avoided completely using the currently available FFF technology, decreased the strength of the 3D parts. Additionally, the properties are directly influenced also by the degree of adhesion between the layers. The fusion lines formed through layer-by-layer deposition can act as frailty lines that could lead to the delamination of 3D printed parts during the testing. 

However, the TS and TM values of 3D printed specimens using PA6/10 wt. % TB N990 (45.8 and 2795 MPa), PA6/20 wt. % TB N990 (38.5 and 2485 MPa) and PA6/10 wt. % TB N990/10 wt. % CF (53.9 and 3641 MPa) are higher than the ones of the specimens printed using a commercial PA6/CF filament (33.9 and 2230 MPa). In terms of elongation at break, slight differences between the 3D printed PA6-based parts and the injected ones can be observed. In terms of Izod impact strength, the values obtained for injected parts and 3D printed parts were almost similar in all the cases and varied from 2.7 to 4.5 kJ/m^2^. The TS and TM values of 3D printed specimens using ABS/10 wt. % TB N990 (29.5 and 2228 MPa) and of ABS/20 wt. % TB N990 (20.5 and 2162 MPa) are fairly similar to ones of the specimens printed using a commercial ABS filament (31.2 and 1582 MPa). The specimens printed using ABS/10 wt. % TB N990/10 wt. % CF/Add filaments presented lower mechanical performance due to the elastomer content in their formulation. The 3D printed specimens obtained from ABS composites and hybrids presented similar elongation at break values and a slightly higher IS performance than their injection-molded counterparts. Overall, the filaments developed in this work based on PA6 and ABS composites and hybrids containing TB N990 and CF, are competitive compared to similar filaments commercially available.

**Figure 14:**
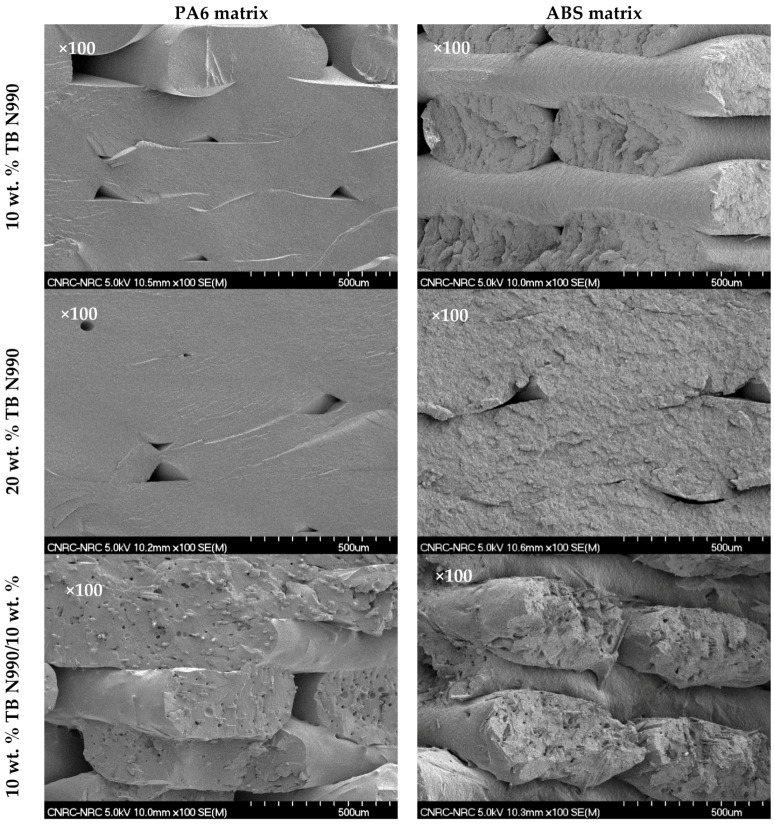
SEM morphology (x100) of 3D printed parts based on PA6 matrix (1st column) and ABS matrix (2nd column) and containing 10 wt. % TB N990 (1st row), 20 wt. % TB N990 (2nd row) and 10 wt. % TB N990/10 wt. % CF (3rd row).

The morphology, as seen by SEM, of 3D printed parts based on PA6 (1st column) and ABS (2nd column) containing 10 wt. % TB N990 (1st row), 20 wt. % TB N990 (2nd row), and 10 wt. % TB N990/10 wt. % CF (3rd row) can be observed in Figure 14. The images shown were taken on fractured surfaces resulting from Izod impact tests. All composites showed varying degrees of layer-to-layer adhesion and interlayer voids. The interlayer voids, as mentioned before, are typically observed in 3D printed parts produced using the FFF technology.

## 3. Material and Methods 

### 3.1. Materials 

The PP used in this work was the Pro-fax PD702 (Lyondell Basell Industries, Long Beach, CA, USA), a homopolymer grade recommended for injection molding applications. The properties of commercial PP compounds having automotive approvals were used for comparison purposes. This commercial PP was the Accutech 40L, a PP filled with 40 wt. % talc, manufactured by ACLO Compounders Inc. (Cambridge, ON, Canada). A coupling agent (CA) was used in selected formulations of PP composites and hybrids. For confidentiality purposes, its name and the concentration will be not disclosed. 

The PA6 used in this work was the Ultramid B27 (BASF Corporation, Budd Lake, NJ, USA), an injection molding grade. The properties of a commercial grade PA6 composite having automotive approvals were used for comparison purpose. It was the Ultramid B3M6 (BASF Corporation), a mineral filled high impact grade containing 30 wt. % minerals (the minerals are not disclosed by the manufacturer). 

The PPS used in this work was the Fortron 0214 (Celanese Corporation, Fortron Industries LLC., Wilmington, NC., USA), a grade recommended by the supplier for injection molding and extrusion applications. The properties of one commercial grade of PPS compound were used for comparison purposes. It was a PPS fiberglass reinforced and mineral filled hybrid, the Fortron 6450A6 (Celanese Canada Inc.).

The ABS used to produce composite and hybrid filaments for 3D printing was the Lustran^®^ Elite 1827 (Ineos Styrolution, Sarnia, ON, Canada), an injection molding grade for high-heat applications. It provides high heat resistance, low gloss, toughness, and easy flow for processing molded colored parts. 

The TB and CB used in this work were Thermax^®^ N990 (TB N990) and Tokai^®^ N762 (CB N762), both supplied by Cancarb (Medicine Hat, AB, Canada). The characteristics for pristine TB N990 are disclosed in Table 10 and for pristine CB N762 are disclosed in Table 11. The CB N762 was used only in one formulation based on each thermoplastic matrix for comparison purposes. 

The CFs used in this work were supplied by Zoltek Corporation (Toray Group, Bridgetown, MO, USA). The CF grade used in PP and PPS hybrids was the Panex^®^ 35 chopped fiber, type 65 having 2.75% PP-based sizing, 6 mm in length and a diameter of elementary fibers of 7.6 μm. The CF grade used in PA6 hybrids was the type 45 having 2.75% PA6-based sizing, a length of 6 mm and a diameter of elementary fibers of 7.1 μm. 

### 3.2. Processing Methods

#### 3.2.1. Extrusion Process

**Figure 15:**
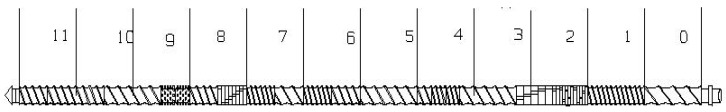
Screw configuration used in extrusion.

The extrusion line used to compound the thermoplastic composites and hybrids was a Leistritz 34 mm co-rotating twin-screw extruder Leistritz Advanced Technologies Corp. BU Extrusion Technology, Somerville, NJ, USA) having 12 mixing zones and L/D ratio of 40. The screw configuration was designed to ensure adequate dynamics in the barrel with the purpose to uniformly disperse and distribute the filler particles in the polymer melts during compounding (Figure 15). A capillary die of 2 mm in diameter was used at the exit of the extrusion line. The temperature profiles were settled keeping in account the melting temperature of each thermoplastic polymer matrix. PA6, ABS, and PPS were dried before compounding as per the TDS recommendation of the suppliers. The extruded formulations of the TB N990 compounds were previously disclosed in Table 1, of the hybrids are presented in Table 5, and of the compounds extruded for 3D printing application are shown in Table 8. In the case of some composites used in 3D printing, where the obtained filaments were very brittle, an additive (Add) was included at low concentration in the formulation to increase the flexibility and ensure a steady 3D printing process. This additive and its concentration are not disclosed here for confidentiality purposes.

#### 3.2.2. Injection Molding

The compounded pellets were dried and then injection molded using a 34 ton Boy injection molding press. The injection barrel and mold temperatures were adapted as a function the melting temperature of the polymer matrix. Standard specimens were molded according to ASTM D638, ASTM D256, and ASTM D648 for tensile, Izod impact, and heat deflection temperature evaluation, respectively. 

#### 3.2.3. Filament Extrusion

The filaments for 3D printing were extruded with a diameter of 1.75 ± 0.05 mm, which is the standard diameter for most FFF 3D printing devices. The PA6- and ABS-based compounds were appropriately dried prior to their transformation into filaments by extrusion. Filaments having a consistent diameter and ovality were produced using a Brabender extrusion line.

#### 3.2.4. 3D Printing by Fused Filament Fabrication 

The previously extruded filaments were first dried at appropriate temperatures and drying durations before using them in 3D printing of specimens for characterization. The parameters used in 3D printing, such as nozzle diameter, layer thickness, printing temperature, plateau temperature, or others were adapted as a function of the polymer matrix behavior used in each composite and to the behavior of each filament. First, specimens for tensile testing from a single formulation were printed as follows: using a bead orientation of +45°/−45° and a layer thickness of 0.2 mm, a bead orientation of +45°/−45° and a layer thickness of 0.35 mm, and using a bead orientation of 0°/180° and a layer thickness of 0.2 mm. After tensile testing, it was observed that the condition +45°/−45° bead orientation and 0.2 mm layer thickness gave the higher tensile performance. Therefore, these parameters were selected to print all the other specimens using a Prusa i3 MK3S printer (Prusa Research a.s., Praha, Czech Republic) to produce tensile specimens (according to ASTM D638), Izod impact specimens (according to ASTM D256) and heat deflection temperature specimens (according to ASTM D648).

### 3.3. Characterization Methods

#### 3.3.1. Morphology

Scanning electron microscopy (SEM) was carried out on surfaces of fractured composites and hybrids in Izod impact test. A coating of gold/palladium alloy was applied on the specimens prior to the observation. A S-4700 SEM Hitachi microscope (Hitachi High Technologies America, Inc., Schaumburg, IL., USA) was used to investigate the dispersion of particles into the matrices and the interfaces between TB particles and matrices.

#### 3.3.2. Rheology Measurements

The rheological properties of the composites described in Table 8 and Table 9 were evaluated using a rotational Advanced Rheometric Expansion System (ARES) rheometer (TA Instruments - Waters L.L.C., New Castle, DE, USA), with plate-plate geometry in dynamic mode. The plate diameter was 25 mm, the gap was around 1.7 mm and the used deformation was 15%. Frequency sweeps were done to evaluate the complex viscosity over a frequency ranging from 0.1 to 100 rad/s. The samples were dried before testing an adequate time and at adequate temperatures corresponding to each matrix. A N_2_ blanket was used during the testing to minimize the oxidation and maintain a dry environment. The tests were done at 200 °C for PP compounds, at 240 °C for PA6 compounds, and for PPS compounds at 300 °C. Before viscosity evaluation, the composites were tested for thermal stability for 30 min. The PP/TB N990 composites demonstrated an excellent thermal stability during the testing time and at the testing temperature. The PA6/TB N990 and PPS/TB N990 composites, where PA6 and PPS matrices typically present a slight viscosity decrease over time, showed an improvement in thermal stability due to the presence of TB N990 which strengthens with TB N990 concentration.

#### 3.3.3. Mechanical Properties

The tensile testing was carried out according to ASTM D638 at a velocity of 5 mm/min on standard type I dog-bone shaped samples. The tensile modulus (TM), tensile strength (TS), and elongation at break (ε%) were evaluated. A video extensometer was used to determine the elastic modulus. The Izod impact strength (IS) was evaluated according to ASTM D256 using notched specimens and a 2 kg hummer. All reported values are the average of five tests.

#### 3.3.4. Heat Deflection Temperature

The heat deflection temperature (HDT) was measured using an Instron Ceast HDT-3-Vicat (Instron, Norwood, MA, USA). The ASTM D648 was applied as follows: a bar of rectangular cross section was tested in the edgewise position as a simple beam with the load of 0.455 MPa applied at its center. The specimen was immersed under load in a heat-transfer medium which temperature increased by 2 °C/min. The HDT under flexural load was recorded as the medium temperature at which test bars deflected by 0.25 mm.

#### 3.3.5. Electrical Resistivity Measurement

Resistivity measurements, for selected formulations, were done on injection molded discs of 3.2 and 1.5 mm in thickness, and 62 and 24.5 mm in diameter, respectively. Extra specimens were fabricated by compression molding. The discs were pressed between two highly conductive cylindrical gold-plated electrodes under a force of 45.4 kgf. The electrodes of 25.4 mm diameter were connected to an Agilent E3633A DC power supply and an Agilent 34420A micro-ohm meter, both from Agilent Technologies (Santa Clara, CA, USA). To improve the contact surface between the electrodes and the test sample, a conductive carbon cloth was used. The voltage drop for the carbon cloth alone was subtracted from the total measured voltage. The electrical resistance, R, of the sample was obtained by dividing voltage drop by the imposed current intensity. The volume resistivity, ρ, was obtained from the resistance R, the electrode surface area, and the sample thickness using the formula ρ = πRD2/4h where D is the electrode diameter and h is the sample thickness.

#### 3.3.6. Cost Calculation

The composites costs were calculated based on market available prices of each thermoplastic polymer, of the thermal black, of the carbon black, and of the different additives used in composition of materials developed in this work. The energy consumption for the materials drying, compounding, injection molding, 3D printing was not considered in this calculation because would not reflect the energy consumption at the industrial scale. 

## 4. Conclusions

In this work, composites and hybrids based on different thermoplastic polymers, such as PP, PA6, PPS, and ABS, containing different concentrations of a novel eco-filler, the thermal black TB N990, were formulated, compounded, and characterized. Numerous advantages were observed when TB N990 particles were used in thermoplastics to produce materials for injection molding and for 3D printing applications.

Composites based on PP, PA6, and PPS containing from 1 up to 40 wt. % TB N990 were compounded using the same compounding process as currently used for thermoplastic/mineral composites (talc, CaCO_3_, clays, silica, glass beads, etc.). A very good distribution and dispersion of TB N990 particles were observed in each matrix for all the TB N990 concentrations while no percolation of particles was detected. Therefore, all the compounds proved to be electrically resistive, even at 40 wt. % TB N990, contrasting with the effect of furnace carbon black which is known to bring high electrical conductivity in thermoplastics. In terms of rheological behavior, the viscosity of PP, PA6 and PPS compounds containing up to 20 wt. % N990 remained very similar to pristine polymers. These lower viscosities are very advantageous for industrial manufacture scale because of the reduced energy consumption and higher throughput rates. The viscosities were slightly increased when 40 wt. % TB N990 was used in the thermoplastic compounds, which should be recommended further for masterbatch production with the purpose to reduce packaging and transportation costs. Mechanical and thermal properties of the PP and PA6 compounds were not deteriorated with the increasing TB N990 content from 1 up to 40 wt. % and presented equivalent performance to commercial compounds containing talc or other mineral filler currently used, for example, in automotive interior parts fabrication. TB N990 proved also to be a good coloring agent, even at very low concentrations, and to provide a “mirror-like” surface quality of the injection-molded parts. Moreover, due to the low cost of TB N990, around 2 USD/kg, it is very advantageous to use it in compounds based on PA6 (around 7.5 USD/kg) and PPS (around 25 USD/kg) leading to cost reductions of final materials up to 50%. 

Microstructure observations done on hybrid composites containing different combinations of TB N990 and carbon fibers, revealed that a part of TB N990 particles are anchored on the surfaces of the carbon fibers. This phenomenon might be explained by a certain affinity existing between the TB N990 (a carbon-based filler) and the carbon fibers (a carbon-based reinforcement), affinity which leads to a degree of synergism between those carbon-based components. Then, TB N990 particles could possibly strengthen the existing reinforcement effect of the carbon fibers. Hence, when replacing a part of the 20 wt. % carbon fibers in PP, PA6 or PPS composites by 3, 5 or 10 wt. % TB N990, the resulting hybrids preserved the tensile performance, the impact performance, and the HDTs close to 20 wt. % CF composites. Another advantage for the TB N990/carbon fibers hybrids is in terms of their cost. The replacement of a part of the expensive carbon fibers (starting from 25 USD/kg) by the less expensive TB N990 (around 2 USD/kg) can bring significant cost reduction while the final materials preserve important performances. 

Finally, TB N990 compounds and TB N990/CF hybrids based on ABS and PA6 matrices were obtained and characterized in this work with the purpose to apply them in 3D printing technology. Filaments for 3D printing process were produced by extrusion. All the filaments evidenced to have an appropriate flexibility for the 3D printing application excepting the ABS hybrid which proved to be brittle. This issue was solved by the addition in the filament formulation of a low quantity of an elastomer. The filament flexibility was increased in this way and made the filament compatible to 3D printing technology. The filaments developed in this work, based on PA6 and ABS composites and hybrids containing TB N990, demonstrated to be competitive to similar filaments commercially available. Thus, the thermoplastic composites containing TB N990 as filler are recommended for the fabrication of filaments for 3D printing of prototype critical parts (all industries) and for fabrication of industrial tooling, jigs, fixtures, spare small parts (automotive, consumer goods and others). 

## Figures and Tables

**Table 1:** Formulation of extruded compounds containing TB N990 and CB N762.

Matrix	TB N990 wt. %					CB N762 wt. %
**PP**	1	3	5	20	40	5
**PA6**	1	3	5	20	40	5
**PPS**	1	3	5	20	40	5

**Table 2:** Properties (and SD) of PP/TB N990 composites.

	Tensile Strength (MPa)	Tensile Modulus (MPa)	Elongation at Break (%)	Impact Strength (kJ/m^2^)	HDT (°C)	Material Cost ($/lb)
**PP PD702**	31.0	1100	910.0	3.2	88	1.000
**1 wt. % N990**	27.2 (0.7)	1158 (19)	928.3 (1.3)	2.7 (0.6)	96 (1)	0.999
**3 wt. % N990**	28.7 (0.8)	1408 (130)	931.8 (2.2)	3.6 (0.3)	97 (1)	0.997
**5 wt. % N990**	28.9 (0.5)	1459 (51)	932.5 (0.4)	2.5 (0.5)	100 (1)	0.995
**5 wt. % N762**	28.4 (0.4)	1348 (98)	928.8 (0.4)	2.2 (0.5)	98 (2)	0.995
**20 wt. % N990**	27.2 (0.2)	1834 (60)	95.3 (0.2)	2.4 (0.3)	100 (1)	0.981
**40 wt. % N990**	26.1 (0.2)	2717 (133)	3.9 (0.2)	1.0 (0.3)	105 (1)	0.872
**PP/40 wt. % talc** **Accutech HP0334T40L**	**26.0**	**2600**	**12.0**	**3.0**	**125**	**~ 1.000**

**Table 3:** Properties (and SD) of PA6/TB N990 composites.

	Tensile Strength (MPa)	Tensile Modulus (MPa)	Elongation at Break (%)	Impact Strength (kJ/m^2^)	HDT (°C)	Material Cost ($/lb)
**PA6 B27**	80.0	3000	15.0	2.4	160	3.75
**1 wt. % N990**	73.0 (0.6)	2974 (53)	109.5 (63)	3.8 (0.5)	148 (45)	3.72
**3 wt. % N990**	76.5 (1.5)	3295 (60)	39.3 (16)	3.5 (0.6)	189 (1)	3.66
**5 wt. % N990**	77.3 (0.6)	3376 (54)	45.8 (17)	3.6 (0.5)	187 (1)	3.61
**5 wt. % N762**	77.4 (0.5)	3178 (49)	98.4 (54)	2.6 (0.7)	186 (1)	3.61
**20 wt. % N990**	78.2 (0.2)	3687 (95)	12.6 (1.2)	2.6 (0.3)	183 (5)	3.18
**40 wt. % N990**	74.5 (6.2)	4851 (117)	2.7 (0.9)	2.6 (0.1)	181 (2)	2.52
**PA6/30 wt. % mineral** **Ultramid B3M6**	**80.0**	**4600**	**5.0**	**6.4**	**195**	**~ 3.00**

**Table 4:** Properties (and SD) of PPS/TB N990 composites.

	Tensile Strength (MPa)	Tensile Modulus (MPa)	Elongation at Break (%)	Impact Strength (kJ/m^2^)	HDT (°C)	Material Cost ($/ lb)
**PPS**	82.1 (3.0)	3661 (120)	3.3 (0.9)	2.7 (0.1)	167 (16)	12.50
**1 wt. % N990**	72.3 (2.1)	3780 (128)	2.3 (0.1)	2.6 (0.1)	172 (3)	12.38
**3 wt. % N990**	68.6 (4.0)	3819 (37)	2.1 (0.2)	2.5 (0.4)	185 (6)	12.15
**5 wt. % N990**	70.4 (3.7)	4030 (123)	2.1 (0.1)	2.6 (0.1)	190 (2)	11.92
**5 wt. % N762**	72.0 (4.6)	4097 (205)	2.1 (0.2)	2.4 (0.4)	189 (5)	11.92
**20 wt. % N990**	62.0 (7.6)	4994 (144)	1.4 (0.2)	1.5 (0.1)	196 (4)	10.18
**40 wt. % N990**	53.4 (11.1)	7056 (84)	0.8 (0.2)	1.5 (0.4)	217 (6)	7.77
**PPS/fiberglass/minerals** **Fortron** **^®^ 6450A6**	**90**	**10,000**	**1.5**	**6.0**	**~ 220**	**~ 12.50**

**Table 5:** Extruded hybrids containing TB N990 and CF; a coupling agent (CA) was used in PP hybrids.

Matrix	CF/TB N990 wt. %/wt. % (+ CA)				
**PP**	20/0	17/3	15/5	10/10	0/20
	+ CA	+ CA	+ CA	+ CA	+ CA
**PA6**	20/0	17/3	15/5	10/10	0/20
**PPS**	20/0	17/3	15/5	10/10	0/20

**Table 6:** Properties (and SD) of PP, PA6, and PPS/CF/TB hybrid composites.

	TS (MPa)	TM (MPa)	ε (%)	IS (kJ/m^2^)	HDT (°C)
***PP/CF/TB Hybrid Composites***					
**100% PP**	31	1100	910	3.2	88
**PP + 20 wt. % CF**	34 (0.3)	9148 (283)	6.3 (1.3)	3.0 (0.1)	149 (0.1)
**PP + 20 wt. % CF + 5 wt. % CA**	80 (0.6)	8929 (268)	8.3 (0.3)	5.1 (0.3)	156 (0.3)
**PP + 17 wt. % CF + 3 wt. % TB**	37 (0.4)	7913 (299)	4.4 (0.4)	2.7 (0.1)	151 (0.3)
**PP + 17 wt. % CF + 3 wt. % TB + 5 wt. % CA**	74 (0.5)	8047 (176)	8.5 (0.4)	4.8 (0.1)	155 (0.2)
**PP + 15 wt. % CF + 5 wt. % TB**	38 (0.2)	7208 (379)	5.4 (0.2)	2.7 (0.1)	154 (0.1)
**PP + 15 wt. % CF + 5 wt. % TB + 5 wt. % CA**	71 (0.4)	7238 (97)	9.3 (0.2)	4.4 (0.2)	151 (0.3)
**PP + 10 wt. % CF + 10 wt. % TB**	31 (0.3)	5636 (118)	12.4 (1.6)	2.3 (0.1)	140 (0.4)
**PP + 10 wt. % CF + 10 wt. % TB + 5 wt. % CA**	60 (0.3)	5276 (220)	11.1 (0.3)	3.3 (0.3)	152 (0.2)
**PP + 20 wt. % TB**	27 (0.2)	1834 (60)	95.3 (3)	2.4 (0.3)	100 (1.1)
**PP + 20 wt. % TB + 5 wt. % CA**	32 (0.2)	1861 (72)	28.5 (4.8)	0.7 (0.0)	108 (0.2)
***PA6/CF/TB Hybrid Composites***					
**100% PA6**	80	3000	15.0	2.4 (0.2)	160
**PA6 + 20 wt. % CF**	145 (1.8)	11420 (457)	10.9 (0.0)	4.4 (0.3)	216 (0.3)
**PA6 + 17 wt. % CF + 3 wt. % TB**	147 (1.0)	11592 (110)	10.1 (0.2)	3.7 (0.3)	213 (0.1)
**PA6 + 15 wt. % CF + 5 wt. % TB**	135 (0.8)	10284 (273)	10.6 (0.3)	3.7 (0.5)	214 (0.4)
**PA6 + 10 wt. % CF + 10 wt. % TB**	114 (0.8)	8211 (285)	11.2 (0.2)	2.3 (0.2)	210 (0.8)
**PA6 + 20 wt. % TB**	78 (0.2)	3687 (95)	12.6 (1.2)	2.6 (0.3)	183 (4.6)
***PPS/CF/TB Hybrid Composites***					
**100% PPS**	82 (3.0)	3661 (120)	3.3 (0.9)	2.7 (0.1)	167 (15.7)
**PPS + 20 wt. % CF**	151 (14.8)	19994 (1063)	4.3 (0.7)	4.3 (0.3)	274 (0.2)
**PPS + 17 wt. % CF + 3 wt. % TB**	144 (7.7)	17116 (2446)	4.7 (0.4)	3.6 (0.3)	272 (1.0)
**PPS + 15 wt. % CF + 5 wt. % TB**	131 (4.3)	17254 (1146)	3.9 (0.3)	3.5 (0.1)	273 (0.7)
**PPS + 10 wt. % CF + 10 wt. % TB**	105 (3.6)	13449 (1183)	3.9 (0.3)	2.7 (0.1)	269 (0.8)
**PPS + 20 wt. % TB**	62 (7.6)	4994 (144)	1.4 (0.2)	1.5 (0.1)	196 (4.4)

**Table 7:** Estimated materials costs (USD/lb) of composites and hybrids.

Thermoplastic Matrix	CF/TB N990 (wt. %/wt. %)				
	20/0 Composite	17/3 Hybrid	15/5 Hybrid	10/10 Hybrid	0/20 Composite
**PP (0.75 USD/lb)**	2.4	2.15	1.99	1.58	0.8
**PA6 (3.75 USD/lb)**	4.8	4.6	4.4	4.0	3.2
**PPS (12.5 USD/lb)**	11.8	11.6	11.4	11.0	10.2

**Table 8:** Formulation of extruded composites and hybrids containing TB N990 and CF for 3D printing.

Matrix	TB N990 wt. %		TB N990/CF wt. %/wt. %
**PA6**	10	20	10/10
**ABS**	10	20	10/10 + Add

**Table 9:** Performance (and SD) comparison of injection molded (IM) and 3D printed (3DP) parts.

	TS (MPa)	TM (MPa)	ε (%)	IS (kJ/m^2^)
***PA6 composites and hybrids***				
**PA6 pristine, IM**	80.0	3000	15.0	8.9 (2.5)
**PA6/10 wt. % TB, IM**	69.2 (2.7)	3077 (38)	21.0 (9.5)	4.1 (1.0)
**PA6/10 wt. % TB, 3DP**	45.8 (2.1)	2795 (345)	3.4 (0.7)	3.0 (0.9)
**PA6/20 wt. % TB, IM**	74.7 (2.1)	3594 (113)	22.9 (9.3)	2.7 (0.8)
**PA6/20 wt. % TB, 3DP**	38.5 (3.9)	2485 (191)	3.8 (0.8)	3.3 (0.8)
**PA6/10 wt. % TB/10 wt. % CF, IM**	109.9 (0.9)	7550 (208)	4.9 (0.2)	2.4 (0.2)
**PA6/10 wt. % TB/10 wt. % CF, 3DP**	53.9 (3.6)	3641 (567)	5.5 (1.1)	4.5 (1.4)
**PA6/CF, commercial, 3DP**	33.9 (2.7)	2226 (204)	3.3 (0.7)	4.1 (0.8)
**PA6, commercial, 3DP**	42.4 (0.5)	1945 (163)	21.0 (0.6)	12.8 (2.1)
***PA6 composites and hybrids***				
**ABS pristine, IM**	32.8 (0.4)	2070 (42)	10.9 (3.4)	11.5 (0.4)
**ABS/10 wt. % TB, IM**	34.0 (0.7)	2449 (74)	3.3 (1.2)	3.6 (0.3)
**ABS/10 wt. % TB, 3DP**	29.5 (0.6)	2228 (44)	4.3 (0.3)	6.2 (0.7)
**ABS/20 wt. % TB, IM**	36.0 (1.0)	3010 (108)	2.4 (0.2)	1.5 (0.3)
**ABS/20 wt. % TB, 3DP**	20.5 (2.9)	2162 (208)	1.4 (0.5)	2.5 (0.2)
**ABS/10 wt. % TB/10 wt. % CF, IM**	66.1 (1.1)	7552 (171)	2.0 (0.3)	3.0 (0.1)
**ABS/10 wt. % TB/10 wt. %CF/Add, 3DP**	17.5 (0.7)	1731 (113)	2.3 (0.2)	4.3 (0.3)
**ABS, commercial, 3DP**	31.2 (0.9)	1582 (88)	9.9 (0.8)	4.1 (0.8)

**Table 10:** Characteristics of thermal black TB Thermax^®^ N990.

Physico-Chemical Test	Minimum	Maximum	Effective Value
Nitrogen Surface Area (m^2^/g)	7.0	12.0	8.5
Oil Absorption Number (mL/100 g)	35.0	44.0	37.8
pH	9.0	11.0	10.0
Ash Content (%)	-	0.20	0.10
Heat Loss (%)	-	0.10	0.05
Fines Content (%)	-	8.0	3.0
Sieve Residue, 325 mesh (ppm)	-	15.0	2.0
Pellet Hardness, Average (g)	-	30.0	19.0
Toluene Extract (%)	-	0.50	0.12

**Table 11:** Characteristics of carbon furnace black CB Tokai^®^ N762.

Physico-Chemical Test	Minimum	Maximum	Effective Value
Iodine Adsorption Number (g/kg)	22.0	32.0	25.0
Nitrogen Surface Area (m^2^/g)	23.0	33.0	26.6
Oil Absorption Number (mL/100g)	60.0	70.0	64.0
pH	6.0	9.0	7.9
Ash Content (%)	-	0.70	0.17
Heat Loss (%)	-	1.0	0.5
Fines content (%)	-	9.0	4.0
Sieve Residue, 325 mesh (ppm)	-	150	50
Pellet Hardness, Average (g)	-	45	23

## References

[1] OECDiLibrary Nanotechnology and Tyres: Greening Industry and Transport.

[2] Merchant Research & Consulting ltd. Carbon Black (BC): 2020 World Market Outlook and Forecast up to 2029. https://mcgroup.co.uk/researches/carbon-black-bc.

[3] Black Bear Carbon. http://blackbearcarbon.com/.

[4] Long C.L., Nascarella M.A., Valberg P.A. (2013). Carbon black vs. black carbon and other airborne materials containing elemental carbon: Physical and chemical distinctions. Environ. Pollut..

[5] (2019). Volume of Emissions in Carbon Black Processes, Climate Policy Watcher: Emission Factors. https://www.climate-policy-watcher.org/emission-factors/introduction-dpn.html.

[6] Crump E.L. Economic Impact Analysis for the Proposed Carbon Black Manufacturing NESHAP, (2000). https://www3.epa.gov/ttnecas1/regdata/EIAs/carbonblackeia.pdf.

[7] Patnaik T., Brown B. (2010). Carbon black: Why quality matters. Rubber Plast. News.

[8] Waste Heat Recovery Power Plant. http://www.cancarb.com/docs/pdf/Waste_Heat_Recovery_Power_Plant.pdf.

[9] Kiraly A., Ronkay F., Berecz T., Majlinger K., Orbulov I.N., Szabo P.J. (2013). Development of electrically conductive polymers. Materials Science Forum.

[10] King J.A., Johnson B.A., Via M.D., Ciarkowski C.J. (2009). Electrical conductivity of carbon-filled polypropylene-based resins. J. Appl. Polym. Sci..

[11] Zhou S., Hrymak A., Kamal M. (2017). Electrical and morphological properties of microinjection molded polypropylene/carbon nanocomposites. J. Appl. Polym. Sci..

[12] Chen Y., Yang Q., Huang Y., Liao X., Niu Y. (2018). Synergistic effect of multiwalled carbon nanotubes and carbon black on rheological behaviors and electrical conductivity of hybrid polypropylene nanocomposites. Polym. Compos..

[13] Gong T., Peng S.-P., Bao R.-Y., Yang W., Xie B.-H., Yang M.-B. (2016). Low percolation threshold and balanced electrical and mechanical performances in polypropylene/carbon black composites with a continuous segregated structure. Compos. Part. B Eng..

[14] Zhansakova K.S., Mitryaeva N.S., Strizhak E.A. Study of the effect of carbon black pigment grades on properties of polypropylene-based composites. Presented at AIP Conference Proceedings.

[15] Spahr M.E., Rothon R., Palsule S. (2016). Carbon Black as a Polymer Filler. Polymers and Polymeric Composites: A Reference Series.

[16] Wang M.-J., Gray C.A., Reznek S.A., Mahmud K., Kutsovsky Y., Seidel A. (2004). Carbon black. Kirk-Othmer Encyclopedia of Chemical Technology.

[17] Kanbur Y., Kucukyavuz Z. (2009). Electrical and mechanical properties of polypropylene/carbon black composites. J. Reinf. Plast. Compos..

[18] Liu Y., Xu W., Zhu J., Wang C., Sheng S. (2015). Polyamide 6/modified carbon black nanocomposites prepared via in situ polymerization. J. Macromol. Sci. Part. B Phys..

[19] Zhang X., Wu W., Liu J., Shen W. (2019). Effect of carbon black self-networking on surface-morphological and electrical properties of immiscible polypropylene/polyamide 6 blends. Mater. Res. Express.

[20] Zhang X., Liu J., Wang Y., Wu W. (2017). Effect of polyamide 6 on the morphology and electrical conductivity of carbon black-filled polypropylene composites. R. Soc. Open Sci..

[21] Sun X., Yu Q., Shen J., Gao S., Li J., Guo S. (2013). In situ microfibrillar morphology and properties of polypropylene/polyamide/carbon black composites prepared through multistage stretching extrusion. J. Mater. Sci..

[22] Garmabi H., Naficy S. (2007). Developing electrically conductive polypropylene/polyamide6/carbon black composites with microfibrillar morphology. J. Appl. Polym. Sci..

[23] Zhang J., Yang B., Fu F., You F., Dong X., Dai M. (2017). Resistivity and its anisotropy characterization of 3D-printed acrylonitrile butadiene styrene copolymer (ABS)/carbon black (CB) composites. Appl. Sci..

[24] Jayanth N., Senthil P. (2019). Application of 3D printed ABS based conductive carbon black composite sensor in void fraction measurement. Compos. Part. B Eng..

[25] Martinez Borja A.L., Perez Bueno J.d.J., Mendoza Lopez M.L. (2018). Composite materials with graphenic materials by extrusion for 3D printing. Mrs Adv..

[26] Mazzanti V., Malagutti L., Mollica F. (2019). FDM 3D Printing of Polymers Containing Natural Fillers: A Review of their Mechanical Properties. Polymers.

[27] Cancarb website - Applications. http://www.cancarb.com/thermal-carbon-black/applications/index.html.

[28] Darmstadt H., Cao N.-Z., Pantea D. (1998). Reactivity and Chemistry of Thermal Carbon Blacks in Comparison to Furnace Carbon Blacks-report.

[29] Pantea D., Darmstadt H., Kaliaguine S., Roy C. (2003). Electrical conductivity of conductive carbon blacks: Influence of surface chemistry and topology. Appl. Surf. Sci..

[30] Tchoudakov R., Breuer O., Narkis M. (1996). Conductive Polymer Blends with Low Carbon Black, Loading: Polypropylene/Polyamide. Polym. Eng. Sci..

[31] Mackay M., Dao T., Tuteja A., Ho D.L., Van Horn B., Kim H.C., Hawker C.J. (2003). Nanoscale effects leading to non-Einstein-like decrease in viscosity. Nat. Mater..

[32] Patti A. (2014). Molecular Dynamics of Spherical Nanoparticles in Dense Polymer Melts. J. Phys. Chem. B.

[33] Parvin N., Ullah S., Mina F., Gafur A. (2013). Structures and mechanical properties of talc and carbon black reinforced high density polyethylene composites, effects of organic and inorganic fillers. J. Bangladesh Acad. Sci..

[34] Liu K., Stadlbauer W., Zitzenbacher G., Paulik C., Burgstaller C. Effects of surface modification of talc on mechanical properties of polypropylene/talc composites. Presented at AIP Conference Proceedings 1713, 31th International Conference of the Polymer Processing Society, ICC Jeju.

[35] Szeluga U., Kumanek B., Trzebicka B. (2015). Synergy in hybrid polymer/nanocarbon composites. A review. Compos. Part A.

